# Back in Time for Breakfast: An Analysis of the Changing Breakfast Cereal Aisle

**DOI:** 10.3390/nu13020489

**Published:** 2021-02-02

**Authors:** Emilie Croisier, Jaimee Hughes, Stephanie Duncombe, Sara Grafenauer

**Affiliations:** 1 School of Human Movement and Nutrition Sciences, University of Queensland, Brisbane, QLD 4072, Australia; e.croisier@uq.edu.au (E.C.); s.duncombe@uq.net.au (S.D.); 2 Grains & Legumes Nutrition Council, 1 Rivett Road, North Ryde, NSW 2113, Australia; j.hughes@glnc.org.au; 3 Children’s Health and Exercise Research Centre, Sport and Health Sciences, College of Life and Environmental Sciences, University of Exeter, Exeter EX1 2LU, UK; 4 School of Medicine, University of Wollongong, Northfields Avenue, Wollongong, NSW 2522, Australia

**Keywords:** breakfast, cereal, sugars, sodium, whole grain, dietary fibre, claims, health star rating

## Abstract

Breakfast cereal improves overall diet quality yet is under constant scrutiny with assertions that the category has not improved over time. This study aimed to comprehensively analyse the category of breakfast cereals, the nutritional values, and health claims across eight distinct sub-categories at four time points (2013, 2015, 2018, and 2020). An audit of products from four major supermarkets in metropolitan Sydney (Aldi, Coles, IGA, and Woolworths) collected ingredient lists, nutrition information, claims and Health Star Rating (HSR) for biscuits and bites; brans; bubbles, puffs, and flakes; granola and clusters; hot cereal flavoured; hot cereal plain; muesli; breakfast biscuits. The median (IQR) were calculated for energy, protein, fat, saturated fat, carbohydrate, sugars, dietary fibre, and sodium for comparisons over time points by nutrient. Data from 2013 was compared with 2020 (by sub-category and then for a sub-section of common products available at each time point). Product numbers between 2013 (*n* = 283) and 2020 (*n* = 543) almost doubled, led by granola and clusters. Whole grain cereals ≥ 8 g/serve made up 67% of products (↑114%). While there were positive changes in nutrient composition over time within the full data set, the most notable changes were in the nutrition composition of cereals marketed as the same product in both years (*n* = 134); with decreases in mean carbohydrate (2%), sugar (10%) and sodium (16%) (*p* < 0.000), while protein and total fat increased significantly (*p* = 0.036; *p* = 0.021). Claims regarding Dietary Fibre and Whole Grain doubled since 2013. Analysis of sub-categories of breakfast cereal assisted in identifying some changes over time, but products common to both timeframes provided a clearer analysis of change within the breakfast category, following introduction of HSR. Whole grain products were lower in the two target nutrients, sodium and sugars, and well-chosen products represent a better choice within this category.

## 1. Introduction

Breakfast and breakfast cereal consumption has been associated with improved diet quality and cereals can significantly contribute to daily micronutrient intake through methods of fortification [1,2,3]. The importance of regular breakfast consumption is emphasised to help adhere to nutritional recommendations and healthy dietary patterns associated with improved health outcomes, such as decreased body mass index (BMI), cardiovascular risk, and enhanced cognitive function [4,5,6]. These outcomes have been examined for Australian children [7] and adults [8] and included more favourable daily nutrient intakes across both age groups in terms of dietary fibre, calcium, and iron among other positive effects related to regular consumption.

Breakfast cereal options have expanded over the years to cater to a growing demand and are often marketed as a convenient source of nutrient-rich grain foods [9]. According to the 2011–2012 National Nutrition and Physical Activity Survey (NNPAS), 36% of Australians (aged 2 years and above) consumed breakfast cereals, and the data suggests that a higher proportion of consumers are in the younger (2–8 years) and older (71 years and over) population groups [10]. Cereal products, milk, and fruit are reportedly the main foods consumed for breakfast by the majority of Australian adults and there is recent data (2020) to suggest that the portion of Australian males (<45 years) consuming breakfast cereal (50%) may have increased over the past two decades [11] with 51 g the median daily amount for males and 35 g for females [10].

There are collective national efforts between the government, public health, and industry sectors to enhance the “nutritional profile of the food supply” [12] using regulatory approaches through to initiatives to guide consumers, and the 2013 to 2020 timeframe was a period of rapid development in this area. Front-of-pack food labelling initiatives, most recently, the 2014 Health Star Rating (HSR) system (an Australian Government initiative), uses a ½ star to 5 star rating to provide a rapid and standardised way of evaluating the overall nutritional profile of packaged foods. The algorithm is based on the presence of positive (fruit/nut/vegetable/legume, dietary fibre, and protein) and risk nutrients (saturated fat, sodium, sugar, and energy (kilojoules) in the food [13,14]. The Healthy Food Partnership Reformulation Program, launched in 2015, provides targets for risk nutrients such as sugar and sodium, with the overarching aim to encourage healthy eating and dietary habits via voluntary product reformulation [12]. As breakfast cereals are commonly consumed and can account for 2.1% of sodium and 2.9% of free sugars in the Australian population (2+ years), they are a focus category [15]. The proposed 2022 target for sodium is a “maximum target of 360 mg/100 g, while for sugar, a 10% reduction is advised for products containing over 25 g sugar/100 g, and a reduction in sugar to 22.5 g/100 g for products between 22.5 and 25 g sugar/100 g” [15]. In regard to nutrition and health claims on food labels, in 2013, the Grains & Legumes Nutrition Council (GLNC), a not-for-profit charity, launched a voluntary Code of Practice for Whole Grain Ingredient Content Claims (hereafter referred to as “The Code”) with guidance claims at ≥8 g (contains), ≥16 g (high in whole grain), and ≥24 g whole grain/manufacturer serve (very high in whole grain). Following that, in 2016, Standard 1.2.7 of the Food Standards Code (FSC) became mandatory and outlines criteria for making nutrition and health claims on food products.

Previous research of the breakfast cereal category was conducted following the introduction of the Daily Intake Guide in 2006 (replaced by HSR in June 2014) and suggested that there had been no change or improvement in breakfast cereals between 2004 and 2010 [16]. A further study of products between 2010 and 2013 was conducted evaluating the impact of the Food and Health Dialogue sodium reformulation, but muesli, hot cereals and breakfast biscuits were excluded [17]. Others have focused on children’s ready-to-eat cereal products, rather than the whole category and only at a single time point [18,19] the latter capturing data from five countries including Australia, New Zealand, the UK, Canada, and the US. The most recent analysis of Australian Breakfast Cereals (data from April 2018) did not include a nutritional evaluation of the category but focused only on claims [20]. This study aimed to comprehensively review the category and compare the nutritional values and claims across eight sub-categories of breakfast cereals over eight years at four time points. Additional analysis with matched products (products with the same name) from 2013 to 2020 was necessary to examine change more precisely.

## 2. Materials and Methods

An audit of the breakfast cereal category from four major Australian supermarkets (Woolworths, Coles, Aldi, and IGA) in metropolitan Sydney was undertaken in 2013, 2015, 2018, and most recently in September 2020. Approximately 79% of the Australian market share is represented by these supermarkets [16] and were therefore chosen to reflect the options available within the breakfast aisle for the Australian population. This recognised process has been outlined in previously published research [21] and the same process was utilised at each data collection point. With permission from store managers, smartphones were used to capture all information on food packaging, including ingredients lists, nutrition information panel (NIP), nutrition and health claims, and HSR (for products collected from 2015). A supplementary internet search was conducted through supermarket websites and identified manufacturer websites using key word searches to ensure all products were collected. Products excluded included breakfast beverages as these are compositionally very different to cereal grain choices.

As a number of products were sold in multiple supermarkets, each product was only recorded once, consistent with previous research [21]. For products that had multiple pack sizes, the largest pack size was used for data analysis as it was more likely to display a greater number of claims. Breakfast cereals in a variety pack were recorded separately, provided each product variety had a separate NIP and ingredients list. In this case, each product variety would have identical on-pack claims.

Following data collection, all breakfast cereals were assigned to one of eight categories using an adapted categorisation system developed through published research [16], outlined in Table 1.

All claims displayed on breakfast cereals in 2013, 2015, 2018, and 2020 were categorised as either nutrition content, general level or high-level health claims as outlined in Standard 1.2.7 of the FSC [13]. Nutrition content claims were defined as statements related to the presence or absence of nutrients or food components in a food product. General level health claims “refer to the nutrients or food and its effect on health”, while high level claims “refer to the nutrients or food and its relationship to a serious disease or biomarker of serious disease” [12]. Eligibility for products to make nutrition content claims was also assessed for the 2020 data, in line with Standard 1.2.7 of the FSC and The GLNC Code for whole grain containing products, as well as proportion of products meeting the Healthy Food Partnership proposed reformulation targets for sugar and sodium reduction [15]. As per the Healthy Food Partnership eligibility criteria, plain ready-to-eat breakfast cereals (e.g., 100% cereal grains, plain puffed grains, processed bran, oat bran, oats, and wheat germ), porridge, breakfast biscuits, and untoasted muesli were excluded from this analysis.

The products collected in 2020 were categorised based on the whole grain content and eligibility for registration with The Code [22] was used to classify products as whole grain or non-whole grain to allow for nutrient comparison between the two categories. Cereals were classified as whole grain if the product contained ≥8 g of whole grain per manufacturer serve and non-whole grain if the product contained <8 g of whole grain per manufacturer serve. Nut and/or seed-based breakfast cereals (e.g., paleo granolas or breakfast biscuits) were excluded from the whole grain and non-whole grain nutrient comparison.

### Statistics

Data from photographs taken at each timeframe were transcribed into a Microsoft^®^ Excel^®^ spreadsheet (Microsoft 365 MSO Version 16.0.13426.20306, Redmond, WA, USA) for analysis. Data was independently checked by a second reviewer for any errors and cross-checked against Mintel Global New Product Database [23]. Information for the data entry included the NIP per serve and per 100 g, ingredients, whole grain content, HSR and nutrition, and health claims, including whole grain, protein, dietary fibre, saturated fat, sugar, and sodium. Health Star Rating was recorded from 2015 to 2020. All data were checked for normality using the Shapiro–Wilk test (IBM SPSS^®^, version 25.0, IBM Corp., Chicago, IL, USA) and the median and interquartile range were presented. A Kruskal–Walis one-way ANOVA with Bonferroni correction for multiple test (IBM SPSS^®^, version 25.0, IBM Corp., Chicago, IL, USA) was used to identify differences (1) in the eight breakfast cereal categories (biscuits and bites; brans; bubbles, puffs and flakes; muesli; granola and clusters, breakfast biscuits; hot cereal plain; hot cereal flavoured) and (2) the within-group differences across the nutrient criteria. A further analysis of matched products with the same name were identified at both the 2013 and 2020 time points in order to assess change in energy and nutrients over time using a paired Mann–Whitney U-test. A Mann–Whitney U test was used to examine the differences between nutrients in whole grain and non-whole grain products, defined according to each product’s eligibility for registration with The Code (≥8 g whole grain per manufacturer serve) [22]. Missing values for dietary fibre were expected, as these are not included on packaging unless claimed, so this was analysed separately. Not all products displayed the HSR, so this was also analysed separately.

## 3. Results

Following the removal of duplicates, data from 1707 breakfast cereals, including biscuits and bites (*n* = 108), brans (*n* = 71), bubbles, puffs, flakes (*n* = 453), granola and clusters (*n* = 427), flavoured hot cereal (*n* = 157), plain hot cereal (*n* = 162), muesli (*n* = 251), and breakfast biscuits (*n* = 78) were collected from 86 food manufacturers/importers between 2013 and 2020. 

The total number of products available on the market had nearly doubled from 2013 (*n* = 283) to 2020 (*n* = 543). Categories such as granola and clusters (28.6%); bubbles, puffs, and flakes (23.1%); and muesli (14.7%) accounted for the majority (66.5%) of products available in 2020, respectively. Comparatively, in 2013, bubbles, puffs, and flakes (35.6%) contributed the most, followed by granola and clusters (16.6%) and muesli (12%). From 2013 to 2020, product numbers had increased within each category, with the exception of biscuits and bites and brans which remained the same or decreased, respectively. Notably, the number of granola and cluster products had more than tripled from 2013 to 2020. Prior to 2015, data was not collected for the category of breakfast biscuits. 

Comparisons were made within each of the eight categories across the eight years at four time points (Shown in Table 2) and compared energy, nutrients and HSR across all eight breakfast cereal categories (per 100 g).

### 3.1. Energy

In 2020, median energy per 100 g across all categories was approximately 1620 kJ, ranging from 1450 kJ to 1820 kJ, with brans (1450 kJ) and biscuit and bites (1490 kJ) providing the least amount of energy compared to breakfast biscuits (1800 kJ) and granola and clusters (1820 kJ) providing the most. Biscuits and bites significantly decreased in energy (−26 kJ from 2013 to 2020) (*p* = 0.047) while energy significantly increased in bubbles, puffs, and flakes (*p* = 0.018); granola and clusters (*p* = 0.000); and hot flavoured cereal (*p* = 0.020), by 23.5 kJ, 30 kJ, and 70 kJ, respectively. As a result, analysis using a Kruskal–Wallis one-way ANOVA with Bonferroni correction demonstrated granola and clusters were significantly higher in energy (kJ) when compared with brans (*p* = 0.002); biscuits and bites (*p* = 0.000); bubbles puffs, and flakes (*p* = 0.000); hot flavoured cereal (*p* = 0.003); hot plain cereal (*p* = 0.000); and breakfast biscuits (*p* = 0.000). Muesli ranked third highest in energy and this resulted in significant differences in comparison to biscuits and bites (*p* = 0.009); and bubbles, puffs, and flakes (*p* = 0.000) which were lower and breakfast biscuits (*p* = 0.003) which were higher (ranking second). Hot flavoured cereal was significantly lower in energy than bubbles, puffs, and flakes (*p* = 0.021).

### 3.2. Protein

**Figure A1:**
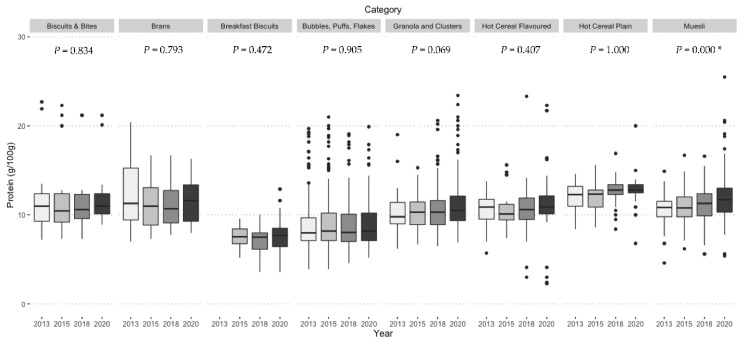
Protein content of breakfast cereals (g/100 g) between 2013, 2015, 2018, and 2020; *p* value comparing 2013 vs. 2020 (2015 vs. 2020 for breakfast biscuits). * Statistically significant *p* < 0.05.

Median protein content of the 2020 breakfast cereals was 10.5 g/100 g with hot plain cereal providing the most (12.8 g) compared to breakfast biscuits (7.7 g) with the least. Almost forty percent of all cereals were considered a “source of protein” containing ≥5 g/serve (Table 3). Over time, protein content has been fairly stable (Appendix A Figure A1) with only hot plain cereal and muesli significantly increasing reported protein (*p* = 0.046 and *p* = 0.014, respectively). Protein was significantly higher in brans (*p* = 0.043), biscuits and bites (*p* = 0.000), and hot plain cereal (*p* = 0.003) but lower in bubbles, puffs, and flakes (*p* = 0.000) and breakfast biscuits (*p* = 0.000) compared to granola and clusters. Hot flavoured cereals was significantly lower than hot plain cereal (*p* = 0.041) and biscuits and bites (*p* = 0.000), but higher than bubbles, puffs and flakes (*p* = 0.000), and breakfast biscuits (*p* = 0.000). Muesli was significantly higher than biscuits and bites (*p* = 0.000), bubbles, puffs, and flakes (*p* = 0.000), and breakfast biscuits (*p* = 0.000).

### 3.3. Fat and Saturated Fat

Total fat significantly increased, by up to 2.5 g in half of the categories, including granola and clusters (*p* = 0.000), hot flavoured (*p* = 0.002) and plain cereal (*p* = 0.049), and muesli (*p* = 0.043) between 2013 and 2020. Granola and clusters were found to contain the most fat (16.4 g) and saturated fat (3 g) and was significantly higher in fat when compared to all other categories: brans (*p* = 0.007); biscuits and bites (*p* = 0.000); bubbles, puffs, and flakes (*p* = 0.005); hot flavoured cereal (*p* = 0.000); muesli (*p* = 0.002); hot plain cereal (*p* = 0.015); and breakfast biscuits (*p* = 0.000). Biscuits and bites contributed the least amount of fat (1.8 g) and saturated fat (0.3 g). Over time fat increased reaching significance for granola and clusters, muesli, and hot flavoured cereal. No significant increases in saturated fat were found over time, apart from granola and clusters (*p* = 0.005).

### 3.4. Carbohydrate and Sugars

**Figure A2:**
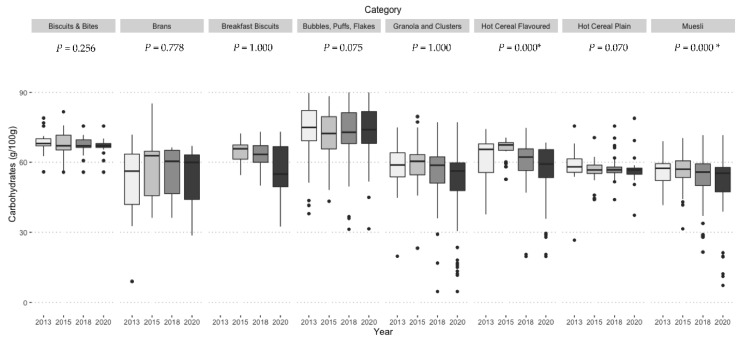
Carbohydrate content of breakfast cereals (g/100 g) between 2013, 2015, 2018, and 2020; *p* value comparing 2013 vs. 2020 (2015 vs. 2020 for breakfast biscuits). * Statistically significant *p* < 0.05.

**Figure A3:**
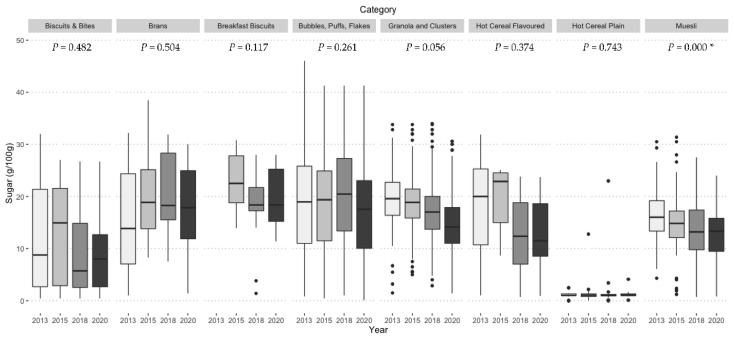
Sugars content of breakfast cereals (g/100 g) between 2013, 2015, 2018, and 2020; *p* value comparing 2013 vs. 2020 (2015 vs. 2020 for breakfast biscuits). * Statistically significant *p* < 0.05.

Carbohydrate content ranged from 55 to 74 g/100 g with an average of 61.6 g/100 g with a difference of 19 g between bubbles, puffs, and flakes, the highest in carbohydrate, to the lowest, breakfast biscuits. Sugars were naturally lowest in hot plain cereals as these often have no added sugars (1 g/100 g) up to 18.4 g/100 g in breakfast biscuits. Hot flavoured cereal and muesli saw a decrease in sugars (*p* = 0.000 and *p* = 0.002) by 8.5 g and 2.7 g, respectively. Breakfast biscuits were significantly higher when compared to bubbles, puffs, and flakes (*p* = 0.003); granola and clusters (*p* = 0.000); muesli (*p* = 0.000); hot flavoured cereal (*p* = 0.002); and hot plain cereal (*p* = 0.000). In 2020, forty one percent of cereals (*n* = 223) contained added dried fruit, consequently only 18% were considered “low in sugar” (≤5 g/100 g) (Table 3). According to the Australian Bureau of Statistics definition of a discretionary breakfast cereal [24] (>30 g sugar per 100 g or >35 g sugar per 100 g with added fruit), 5% (*n* = 15/283) of all cereals were considered discretionary in 2013, compared to only 3% (*n* = 17/543) of cereals in 2020. In comparison to other nutrients, carbohydrate has reduced over time (Appendix B Figure A2), reaching significance in granola and clusters, hot flavoured cereal, muesli, and breakfast biscuits which was reflected in significant reduction in sugars (Appendix C Figure A3) and in particular, a 5.4 g reduction for granola and clusters (*p* = 0.000), 8.5 g reduction for hot flavoured cereal (*p* = 0.000), 2.7 g reduction for muesli (*p* = 0.002); the exception was breakfast biscuits, where although carbohydrate was reduced, sugars did not reach significance.

### 3.5. Dietary Fibre

The average amount of dietary fibre across all categories was 10 g/100 g, ranging from breakfast biscuits and bubbles, puffs, and flakes contributing the least (7.2–7.4 g) up to the most, in brans (16.3 g). Overall, 84% of cereals were at least a “source of fibre” (≥2 g/serve) (Table 3). Bubbles, puffs, and flakes were significantly lower than brans (*p* = 0.049), granola and clusters (*p* = 0.000), hot flavoured cereal (*p* = 0.000), muesli (*p* = 0.000), and hot plain cereal (*p* = 0.000), while breakfast biscuits were significantly lower than both granola and clusters (*p* = 0.009) and muesli (*p* = 0.019). For the majority of categories, there were no significant changes seen in dietary fibre between 2013 and 2020; however, breakfast biscuits increased slightly (0.7 g) from 2015 to 2020 (*p* = 0.034).

### 3.6. Sodium

**Figure A4:**
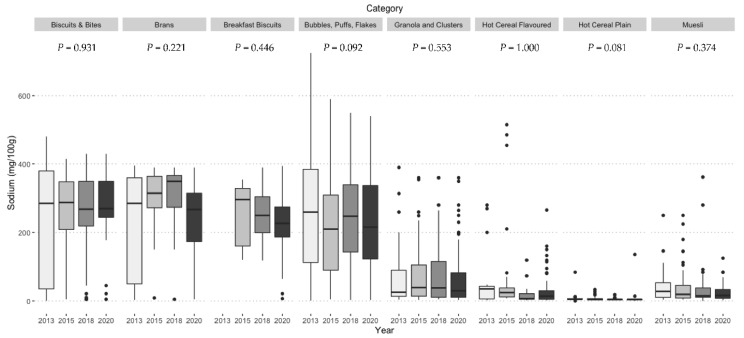
Sodium content of breakfast cereals (g/100 g) between 2013, 2015, 2018, and 2020; *p* value comparing 2013 vs. 2020 (2015 vs. 2020 for breakfast biscuits). * Statistically significant *p* < 0.05.

There were no significant differences found between categories for sodium; however, hot plain cereal provided the least with 5 mg/100 g and biscuits and bites and brans provided the most at 270 mg/100 g and 267.5 mg/100 g, respectively. Two thirds of all breakfast cereals were considered “low in sodium” (≤120 mg/100 g) (Table 3). Sodium improved or stayed the same across the majority of categories, except for granola and clusters. However, the only statistically significant change was seen in hot flavoured cereal (*p* = 0.000) which decreased by 21 mg from 2013 to 2020 (Appendix D Figure A4).

### 3.7. Micronutrients

Micronutrient fortification was found in 23.4% (*n* = 127) of total products in 2020. Minerals such as calcium carbonate, iron, and zinc oxide were common additions, while vitamins such as niacin, vitamin B6, riboflavin, folic acid, thiamin, vitamin D, and vitamin E were found to be fortified in these products. Brans had the highest number of fortified products in their category (81.3%), followed by biscuits and bites (72%); bubbles, puffs and flakes (63.5%); breakfast biscuits (22.6%); and granola and clusters (4.5%).

### 3.8. Health Star Rating

The HSR was featured on almost three-quarters of breakfast cereals (73%, *n* = 397). Of these, 84% were rated between 4 and 5 stars, 9% were between 3 and 3.5 stars and only 6% had a HSR ≤ 2. The average HSR across all categories was 4.06 and ranged from brans, at 3.5 up to hot plain cereal rated the highest at 5. The remaining categories reported a median HSR of 4. Data from 2013 was not included as the HSR system commenced in 2014. The HSR decreased for some of the categories from 2015 to 2020, and this included biscuits and bites (*p* = 0.020), brans (*p* = 0.020), and muesli (*p* = 0.000). Conversely, breakfast biscuits increased in their HSR from 3 (2015) to 4 (2020) (*p* = 0.000).

### 3.9. Comparison of Matched Products from 2013 to 2020 Products (n = 134) 

The nutrient composition of breakfast cereals marketed under the same name in 2013 and 2020 were further analysed to ensure a fair assessment of change over time (Table 4). Only one quarter of cereals collected in 2020 were also available on shelf in 2013 (*n* = 134). Since 2013, there was notable change in the nutrition composition of cereals marketed as the same products in both years; mean carbohydrate (2%), sugar (10%), and sodium (16%) content decreased (*p* < 0.000), while protein and total fat significantly increased (*p* < 0.036; *p* < 0.012). The change in energy, saturated fat, and dietary fibre content did not reach significance. We noted that the median for sodium was significantly different between the time periods but in the opposite direction, and this was due to a number of higher sodium products, up to 725 mg/100 g available in 2013 which have now decreased. The number of whole grain products (containing ≥ 8 g whole grain per serve) increased from 67% (*n* = 86) to 74% of the sample (*n* = 99) between 2013 and 2020. Whole grain content increased from 53% in 2013 to 57% in 2020 within the identical group of products. 

### 3.10. Proposed Healthy Food Partnership Reformulation Targets

Overall, 311 (57%) breakfast cereals met the inclusion criteria for sodium and sugar reformulation. Ninety three percent (*n* = 288) of eligible breakfast cereals met the proposed sodium reformulation target of ≤360 mg/100 g. Regarding sugar, most eligible cereals met the proposed target of <22.5 g sugar/100 g (79%, *n* = 247), while only 6% (*n* = 19) met the proposed sugar reformulation target between 22.5 and 25 g/100 g.

### 3.11. Nutrition and Health Claims

The prevalence of nutrition and health claims were compared across the four time points (Table 5). Dietary fibre and whole grain content claims were reported the most frequently on food products at all time points, and approximately doubled from 2013 (*n* = 168, *n* = 132) to 2020 (*n* = 353, *n* = 283, respectively). Between 2013 and 2020 the largest increase in nutrition claims related to plant-based (e.g., suitable for vegetarians and/or vegans, plant protein, and plant-based diets) increasing from 17 to 177 claims (ninefold increase). Similarly, protein content claims increased substantially from 27 claims in 2013 to 161 claims in 2020 (496% increase). The glycemic index, fat, and sodium/salt claims decreased in the same time period. The top three general level health claims are presented in Table 5. There had been a sevenfold increase in the number of products displaying dietary fibre general level health claims from 2013 (*n* = 7) to 2020 (*n* = 56). Similar to this, general health claims for protein were first reported in 2018 and were found more frequently in 2020 (*n* = 10). The only high-level health claim found on breakfast cereals related to the cholesterol lowering effects of beta glucan found in whole grain oats.

### 3.12. Whole Grain Breakfast Cereals

Products in 2020 were categorised as whole grain based on the GLNC criteria ≥8 g whole grain per manufacturer serve (Table 6). Sixty seven percent of cereals were considered whole grain (*n* = 364), most of which (76%, *n* = 278) contained ≥50% whole grain ingredients. The nutrition information for these products were compared with non-whole grain products (*n* = 179) and found to be significantly higher in protein (*p* < 0.001), total fat (*p* = 0.006), dietary fibre (*p* = 0.009), and lower in carbohydrate (*p* = 0.013), sugars (*p* < 0.001), and sodium (*p* < 0.001) (Table 6). Since 2013, there had been a twofold increase in the number of whole grain cereals on the market (170 products in 2013 vs. 364 products in 2020). 

## 4. Discussion

Although breakfast cereals have been designed to be conveniently ready-to-eat and easy to prepare, a wide variety of products exist within this category from single ingredient oat-based products which may require cooking or the addition of other ingredients (i.e., milk to prepare a porridge-style breakfast dish), through to breakfast biscuits which offer a convenient on-the-go, shelf-stable, energy dense food that requires no preparation. The manner in which these foods are consumed is different, and these differences also extend to the nutrient profile, a point established in earlier research [25]. The heterogeneity in the nutrition composition meant that eight sub-categories were necessary for more accurate comparisons of the nutritional value of breakfast cereals, with significant changes observed within some categories over time, but particularly when a sub-set of data was used with products marketed with the same name between 2013 and 2020.

Improvements in protein, carbohydrate, total sugars, dietary fibre and sodium occurred for the majority of products, with significant (*p* < 0.05) changes seen in muesli products from 2013 to 2020. The HSR and saturated fat values were maintained over time for the majority of categories, while energy (kJ) and total fat content increased for the majority of categories. All categories were rated above a HSR of 3, that is above the score which has been determined as a cut-off for consumers identifying a food to be “unhealthy” [26]. The product categories lowest in total fat (≤3 g/100 g) and saturated fat (≤1.5 g/100 g), biscuits and bites, brans, bubbles, puffs and flakes, tended to be higher in sugars and sodium. Conversely, those higher in fat and saturated fat, including plain hot cereal, flavoured hot cereal, muesli, granolas and clusters, were lowest in sodium. To ensure a fair assessment of change could be made, products available under the same name in 2013 and in 2020 were also compared confirming significant reductions in mean carbohydrate (2%), sugar (10%) and sodium (16%), while protein and total fat had significantly increased. Previous reviews have reported no change to the nutritional composition of this category between 2004 and 2010, although there had been a reduction in sodium in cereals for children, and the authors noted that dried fruit (in ~50% of products) contributed to the classification of cereals being high (>12.5 g/100 g) in sugar (40%) [16]. In contrast, analysis of products from 2010 to 2013 (excluding muesli, hot cereal, and breakfast biscuits) focusing only on sodium, found a 25% reduction from 316 to 237 mg/100 g (*p* < 0.001). The more recent data signals a further improvement in sodium for the category in the 2013 to 2020 timeframe.

Regular breakfast consumption has been shown to improve diet quality, cardiovascular risk factors and is associated with higher intake of micronutrients [27]. Interestingly, breakfast skipping increases risk of Type 2 Diabetes Mellitus, subclinical atherosclerosis, and detrimental cardiometabolic health independent of diet quality [28]. Some improvement in diet quality among breakfast cereal consumers is likely a result of food fortification by manufacturers and is commonly found in ready-to-eat, pre-packaged products. Iron, calcium, zinc, and B vitamins are commonly added, and this is especially important for growing adolescents to help them meet their requirements [29]. A higher prevalence of inadequacy in nutrients such as thiamin, riboflavin, vitamin D, calcium, magnesium, and selenium have been reported in older adults and vitamin D, calcium, magnesium, and selenium inadequacies can occur in younger populations (>19 years above) [30]. Rates ranging from 11 to 30% for micronutrient inadequacies have been reported in European adults regarding certain vitamins and minerals, such as: copper, folate, selenium, iodine, vitamin B12, and vitamin C [30,31]. Deficiencies in these nutrients can result in increased risk of falls, poorer bone density, cognitive decline, and can impede growth and development [32]. A means of improving the intake of these micronutrients can be through consumption of fortified products which is accessible in nearly a quarter (23.4%) of breakfast cereals (2020). These products may be especially important for certain groups such as children and adolescents, vegetarians, and pregnant or breastfeeding women, who have increased nutritional requirements [32]. 

Product reformulation has the aim of manipulating macronutrient and micronutrient composition in foods either to enhance the taste, appearance, consistency, shelf life, or to improve the nutritional quality [33]. In particular, risk nutrients such as sugars and sodium have been perceived contentiously in breakfast cereals [34,35]. The Healthy Food Partnership (HFP) proposed targets for sugars (10% reduction for products containing over 25 g/100 g) and sodium (360 mg/100 g) (unpublished data) can be used as criteria to compare the breakfast cereal products to. Review of all eight categories indicate median values adhered well below the suggested targets; sugars ranging from 1 g/100 g to 18.4 g/100 g (2020) and sodium ranging from 5 mg/100 g to 270 mg/100 g (2020) with 79% meeting the lower (<22.5 g/100 g) sugar target and 93% meeting the sodium target. However, products can only be reformulated to an extent, based on certain manufacturing requirements as some product formats require sodium to form puffs, flakes and hold the integrity of biscuits or bites. In addition to this, salt can also act as a preservation method to extend shelf-life, controlling growth of mould by reducing water activity and maintaining a crisp product [36]. This is seen in the elevated sodium content for bubbles, puffs, and flakes; breakfast biscuits; brans; and biscuits and bites compared to the remaining categories. Despite this, a gradual reduction in sugars and sodium has been a key strategy employed by the industry [37].

The 2014 HSR system is a voluntary front-of-pack labelling initiative that evaluates selected aspects of the nutritional quality of products. For example, muesli is rated lower compared to hot plain cereal products, due to higher values in sodium, sugars, total fat and saturated fat, and energy (kJ). However nutrient values should not be evaluated in isolation without first considering the ingredient list. Higher energy content may be attributed to the addition of nuts and seeds and dried fruit which can both provide a more palatable option and also added nutritional value from its mono- and poly-unsaturated fats and dietary fibre. Coconut is also a popular addition to cereals, increasing dietary fibre, but also increasing energy, fat, and saturated fat. Importantly, whole grain content is excluded from the algorithm and research from products collected in 2018 indicates that less than a star (0.7 stars) differentiates whole grain and refined grain products, raising concerns that dietary fibre may not be a sufficient proxy measure for whole grain foods [38].

Breakfast cereal foods provide an opportunity to consume a rich source of whole grains. Over half of breakfast cereals in 2020 (67%) were categorised as whole grain based on The Code (≥8 g per manufacturer serve), of which 76% were ≥50% whole grain. These products were significantly (*p* < 0.05) higher in protein, total fat, saturated fat, dietary fibre, and significantly lower in carbohydrate, sugars, and sodium. Consumption of products identified as whole grain can assist in meeting the adult’s Daily Target Intake for whole grain of 48 g/day. Australian data suggests that the median consumption of whole grain is below this, at 38 g/day [39], and that intake is inadequate amongst children (median 16.5 g/day) and adults (median 21.2 g/day) with only 27% of individuals (≥9 years) meeting the 48 g Daily Target Intake (DTI) [22,39,40]. Whole grain products can also contain a range of phytochemicals associated with protective cardiometabolic health benefits [41,42] and as such, consuming these breakfast cereal products can provide a rich and unique source of fortified micronutrients and dietary fibre to help meet the suggested dietary targets. In fact, breakfast cereal and bread are known to be the key sources of whole grain in the Australian diet [27] and this would be true of other countries who share a similar dietary pattern. 

Dietary fibre can be sourced from breakfast cereals, particularly whole grain cereals and bran-based products. In Australia, 42.3% of children and 28.2% of adults met the Adequate Intake (AI) for dietary fibre, and <20% of adults met the Suggested Dietary Target (SDT) (25–30 g/day) to reduce the risk of chronic disease as per the Nutrient Reference Values [43]. Dietary fibre is associated with decreased chronic disease risk (cardiovascular and diabetes), bowel cancer, and mortality rates, and can help maintain body weight [44]. Cereal fibre helps alter the gut microbiota composition, and this can have subsequent implications on an individual’s health and well-being [45]. There is increased consumer awareness of day-to-day gut health and the microbiome and these are also important considerations for consumers [16]. There has been a substantial increase in the prevalence of product on pack claims related to dietary fibre and whole grain especially. This may reflect the emerging health trends and priorities for product reformulations. The range and number of breakfast cereal products have also considerably increased over the eight years since 2013 to include products that cater to specialised dietary requirements, such as: gluten free (18.4%) and plant-based (vegetarian, vegan) (33.1%) in 2020. The future of the market is contingent on consumer demand and there is the possibility that greater significance may be placed on aspects such as country of origin and ingredient sourcing; sustainability and plant-based foods; and gut health.

This study is the first of its kind to comprehensively review breakfast cereals’ nutrient composition and health claims in Australia across four time points and captures at least 79% of the market. This study is not without limitations however. Like all food categories, this category is dynamic, with products discontinued and others added, so a comparison over time was accompanied by a more specific analysis of products of the same name available in 2013 that were still on the market in 2020. In particular, we noted a large increase in two categories, hot flavoured cereal and granolas and clusters with almost three times as many products in 2020 as in 2013. Assessing change in breakfast biscuits across time was limited due to data not having been collected prior to 2015. In addition to this, certain nutrients such as dietary fibre are not mandatory to report on the NIP and hence resulted in some missing values. The accuracy of nutritional values collected from the NIP is dependent on the reporting of manufacturers. No separate analysis was conducted to evaluate these nutritional values. We also acknowledge that nutrient based analysis may underplay the efforts made by manufacturers to reformulate products by improving ingredients especially whole grains, nuts, seeds, legumes, and fruit or changes to other more minor ingredients.

## 5. Conclusions

This study provided a unique opportunity to review the composition of breakfast cereal products prior to, and for the six years following the introduction of HSR. We acknowledge that manufacturers may have already made significant changes to the formulation of products in the lead up to 2014, so even this longer-term view is limited in terms of true outcomes for the industry. What is clear, is that the diversity within the category continues to grow, with opportunities for consumers to make good choices. Although a great deal of work has progressed the nutrient profile of the category, the research is also of interest to food technologists who should continue to work towards innovative solutions to improve this further. An increasing number of whole grain products was a positive result of this review, which coincided with the introduction in 2013 of the voluntary Code of Practice of Whole Grain Ingredient Content Claims. Overall, whole grain products were significantly higher in protein, total fat, and dietary fibre and lower in carbohydrate, sugars, and sodium compared to non-whole grain cereals and as such, represent a better choice within the category.

## Figures and Tables

**Table 1:** Classification of Breakfast Cereal categories.

Category	Description of Types
Biscuits and Bites	Includes cereal made from whole wheat formed into a biscuit shape, typically consumed with milk. May be fortified with micronutrients.
Brans	Includes cereal made primarily from wheat bran. May be fortified with micronutrients.
Bubbles, Puffs, Flakes	Includes cereal made from toasted puffed or popped grains or flakes made from corn, wheat, oats and/or rice. May be fortified with micronutrients.
Muesli	Includes raw or dry toasted cereal with “muesli”, “bircher”, or “swiss” in the name of the product, commonly made from rolled oats without the addition of oils. May contain added dried fruit, nuts, seeds, coconut and/or added sugar.
Granola and Clusters	Includes cereal with “granola” or “cluster” in the name of the product, made primarily from rolled oats with added sweeteners and/or oils baked until crisp, toasted, and golden brown. May contain added dried fruit, nuts, seeds and/or coconut.
Hot Cereal, Plain	Includes plain uncooked quick cooking or rolled oats, used to prepare a porridge-style breakfast dish.
Hot Cereal, Flavoured	Includes flavoured uncooked quick cooking or rolled oats, used to prepare a porridge-style breakfast dish.
Breakfast Biscuits	Includes convenient, “on-the-go” single serve biscuits typically made from wheat flour and/or nuts and seeds. Consumed without milk and are typically higher in total fat and sugars.

**Table 2:** Nutrient comparison and Health Star Rating (HSR) per 100 g of breakfast cereals between 2013, 2015, 2018, and 2020 (median ± (IQR)).

Category	Year	Number of Products	Energy (kJ)	Protein (g)	Total Fat (g)	Saturated Fat (g)	Carbohydrate (g)	Sugars (g)	Dietary Fibre (g)	Sodium (g)	HSR
*Biscuits and Bites*	2013	25	1516 (1490–1570)	11 (9.3–12.4)	1.6 (1.4–2.0)	0.4 (0.3–1.0)	68 (67–70.1)	8.8 (2.7–21.4)	10.1 (9–11)	285.0 (35–380)	n/a
	2015	30	1555 (1490–1590)	10.5 (9.2–12.4)	1.8 (1.3–4.8)	0.4 (0.3–1.3)	67.1 (65.1–71.8)	15 (2.9–21.6)	9.3 (7.3–11)	287.5 (200–350)	4.3 (4–4.5)
	2018	28	1490 (1475–1540)	10.6 (9.5–12.4)	1.8 (1.3–3)	0.4 (0.3–0.9)	67 (66.4–69.7)	5.8 (2.5–17.1)	10.5 (8.6–12.1)	268.5 (205.5–350)	4.5 (4–4.8)
	2020	25	1490 (1470–1550)	11 (10.1–12.4)	1.8 (1.3–2.5)	0.3 (0.3–0.6)	67 (66.4–68)	8 (2.7–12.7)	10.5 (8.3–11.1)	270.0 (244–350)	4 (3.5–4.5)
	*p* value		0.047 *	0.834	0.914	0.904	0.256	0.482	0.363	0.931	0.020 *
*Brans*	2013	20	1440 (1380–1520)	11.3 (9.4–15.6)	3 (1.8–5.5)	0.5 (0.5–1.1)	56.2 (41.5–63.9)	13.9 (5.8–25)	16.4 (15.2–30)	285 (43.5–360)	n/a
	2015	19	1420 (1370–1455)	11 (8.9–13.1)	2.7 (1.9–4.5)	0.5 (0.4–1.0)	62.8 (45.8–64.8)	18.9 (13.8–25.2)	15.3 (14.8–28.8)	315 (272.5–364)	4.3 (4–5)
	2018	16	1425 (1380–1450)	10.7 (8.8–12.8)	2.6 (1.85–4.5)	0.5 (0.4–1.0)	60.4 (45–65.3)	18.3 (14.4–28.5)	16.7 (15.2–30.8)	350 (272.5–367.5)	4.8 (4.5–5)
	2020	16	1450 (1365–1515)	11.6 (9.1–13.6)	3.4 (2.7–5.4)	0.7 (0.6–1.0)	60 (43.3–63.6)	17.9 (11.6–26.1)	16.3 (15–30.8)	267.5 (157.5–330)	3.5 (2.5–4.8)
	*p* value		0.617	0.793	0.607	0.432	0.778	0.504	0.904	0.221	0.020 *
*Bubbles*, *Puffs*, *Flakes*	2013	101	1590 (1550–1630)	8 (7.1–9.7)	2.1 (1.3–4.2)	0.5 (0.3–1.2)	74.9 (69.2–82.2)	19 (11–25.8)	7.2 (3.9–9.6)	260 (112–385)	n/a
	2015	120	1585 (1520–1635)	8.2 (7.1–10.2)	2.8 (1.6–5)	0.7 (0.4–1.1)	72.4 (65.8–79.7)	19.4 (11.2–25)	8 (6.6–11)	210 (90–310)	4 (3.5–4.5)
	2018	106	1605.5 (1560–1650)	8.1 (7–10.1)	2.7 (1.5–5)	0.7 (0.4–1.0)	72.9 (67.9–81.3)	20.5 (13.3–27.4)	7.5 (4.1–9.9)	247.5 (141–340)	4 (3–4)
	2020	126	1613.5 (1570–1650)	8.2 (7.1–10.2)	2.5 (1.6–4.6)	0.8 (0.5–1.0)	74 (68–81.8)	17.6 (10–23.1)	7.4 (3.3.–9.1)	216 (120–340)	4 (3–4.5)
	*p* value		0.018 *	0.904	0.124	0.294	0.075	0.261	0.078	0.092	0.283
*Granola and Clusters*	2013	47	1750 (1640–1849)	9.8 (9–11.4)	13.9 (10–17)	2.4 (1.9–3.9)	58.9 (53.9–64.1)	19.6 (16.4–22.8)	9.1 (7.6–10.5)	25 (13.5–90)	n/a
	2015	111	1750 (1659–1820)	10.3 (8.9–11.5)	13.6 (10.2–16.4)	2.4 (1.7–3.8)	60.4 (54.6–63.4)	18.9 (15.9–21.5)	8.2 (7–9.3)	39 (14–105)	4 (4–4)
	2018	113	1800 (1730–1890)	10.3 (8.9–11.6)	15 (12.5–19.2)	2.4 (1.8–3.7)	58.8 (51.1–62.3)	17 (13.7–20)	8.1 (7–9.2)	38 (11–115)	4 (4–4)
	2020	156	1820 (1740–1945)	10.5 (9.4–12.2)	16.4 (13.7–21.8)	3 (2–5.3)	56.3 (47.9–59.9)	14.2 (11–17.9)	8.3 (7.1–10.3)	29.5 (11–84.5)	4 (4–4.5)
	*p* value		0.000 *	0.069	0.000 *	0.005 *	0.000 *	0.000 *	0.151	0.553	0.091
*Hot Cereal Flavoured*	2013	23	1590 (1513–1615)	10.9 (9.6–11.8)	6.7 (6.4–7.8)	1.3 (1.2–1.9)	65.6 (55.6–67.9)	20.0 (10.7–25.3)	8.2 (7.3–12.2)	35 (6–43)	n/a
	2015	27	1618 (1600–1640)	10.1 (9.5–11.2)	6.8 (6.1–7.9)	1.2 (1.2–1.7)	67.4 (65.2–68.4)	22.9 (15–24.6)	7.8 (7.3–8.7)	24 (12–38)	4 (4–4)
	2018	44	1610 (1560–1640)	10.6 (9.5–12)	7.6 (6.5–9.1)	1.3 (1.2–1.8)	62.2 (56.5–66)	12.4 (6.3–18.9)	8.5 (7.1–10.2)	7 (5–21)	4 (4–4.5)
	2020	63	1620 (1600–1640)	10.9 (10.1–12.2)	8.4 (7–11)	1.5 (1.3–1.9)	59.3 (53.6–65.5)	11.5 (8.6–18.7)	9 (7.4–10.7)	14 (5–29.5)	4 (4–4.5)
	*p* value		0.020 *	0.407	0.002 *	0.075	0.000 *	0.000 *	0.367	0.000 *	0.418
*Hot Cereal Plain*	2013	33	1590 (1572–1600)	12.3 (11–13.2)	8.7 (7.8–8.8)	1.6 (1.5–1.7)	58.1 (55.7–61.4)	1.0 (1–1.3)	10.1 (9.7–12.5)	5 (5–7)	n/a
	2015	38	1600 (1572–1650)	12.4 (10.9–12.8)	9 (8.5–9.4)	1.7 (1.5–1.9)	56.7 (55.2–58.9)	1.1 (0.7–1.3)	11.8 (10–12.9)	5 (3–7)	5 (5–5)
	2018	44	1590 (1568.5–1628)	12.8 (12.3–13.4)	8.8 (7.3–9.2)	1.6 (1.2–1.7)	56.8 (55.5–58.1)	1.0 (0.9–1.2)	11.4 (9.4–12.3)	5 (3–5.5)	5 (5–5)
	2020	47	1590 (1570–1620)	12.8 (12.5–13.4)	8.9 (8.5–9.2)	1.5 (1.2–1.7)	56.7 (55–57.6)	1.0 (1.0–1.3)	11.5 (10.2–12.2)	5 (3–5)	5 (4.5–5)
	*p* value		0.361	0.046 *	0.049	0.189	0.701	0.743	0.307	0.081	0.007
*Muesli*	2013	34	1590 (1538–1680)	10.9 ± (9.8–11.6)	9.4 (7–11.4)	1.8 (1.3–2.9)	57.5 (52–59.6)	16.1 (13–19.3)	10.6 (8.3–11.3)	27.5 (10–54)	n/a
	2015	68	1615 (1547.5–1705)	10.8 (9.8–12.1)	10.4 (8–12.9)	1.8 (1.3–2.6)	57.1 (53.4–60.6)	14.9 (12.1–17.3)	10 (8.3–11.1)	19 (8.5–46.5)	4.5 (4–4.5)
	2018	69	1610 (1530–1690)	11.3 (9.9–12.4)	10.1 (7.7–13)	1.9 (1.3–2.7)	55.8 (50–59.4)	13.2 (9.8–17.4)	10.3 (8.1–11.7)	15 (11–38)	4.5 (4–4.5)
	2020	80	1625 (1558.5–1745)	11.7 (10.3–13)	10.9 (8.3–16.6)	1.7 (1.3–3.1)	55.4 (47.4–57.9)	13.4 (9.3–15.9)	10.7 (9.1–12)	16 (8–33.5)	4 (3.3–4.5)
	*p* value		0.265	0.014 *	0.043 *	0.428	0.017 *	0.002 *	0.174	0.374	0.000 *
*Breakfast Biscuit*	2013	0	n/a	n/a	n/a	n/a	n/a	n/a	n/a	n/a	n/a
	2015	16	1855 (1795–1940)	7.6 (6.4–8.5)	15.3 (13.5–21.1)	3.2 (1.5–10)	65.9 (60.3–67.5)	22.6 (18.5–27.9)	6.5 (4.6–7.2)	296.5 (159.5–329)	3 (3–3.3)
	2018	31	1850 (1815–1880)	7.5 (6.2–8)	15.4 (14.5–16.9)	1.7 (1.4–2.1)	63.4 (60–67.1)	18.4 (17.3–21.8)	7.1 (5.2–9.1)	250 (199–305)	3 (3–3.5)
	2020	31	1800 (1695–1885)	7.7 (6.45–8.5)	15.4 (14.5–18.5)	2.4 (1.6–6.7)	55 (49.5–66.7)	18.4 (15.3–25.3)	7.2 (5.4–11.4)	227 (187–274.5)	4 (4–4.5)
	*p* value		0.222	0.472	0.845	0.054	0.006 *	0.117	0.034 *	0.446	0.000 *

* Statistically significant (*p* < 0.05).

**Table 3:** Number and proportion of breakfast cereals meeting claim criteria in 2020 (*n* = 543).

Nutrient Content Claim Eligibility	2020 Breakfast Cereals *n* (%)
Eligible for WG claim (≥8 g/serve)	364 (67)
Contains WG (≥8 to <16 g/serve)	50 (9)
High in WG (≥16 to <24 g/serve)	76 (14)
Very high in WG (≥24 g/serve)	238 (44)
Low Fat (≤3 g/100 g)	106 (20)
Low Saturated Fat (≤1.5 g/100 g)	247 (45)
Source of protein (≥5 g/serve)	212 (39)
Good source of protein (≥10 g/serve)	14 (3)
Low sugar (≤5 g/100 g)	100 (18)
Low sodium (≤120 mg/100 g)	360 (66)
Source of fibre (≥2 to <4 g/serve)	214 (39)
Good source of fibre (≥4 to <7 g/serve)	194 (36)
Excellent source of fibre (≥7 g/serve)	47 (9)

**Table 4:** Comparison of nutrient content per 100 g for breakfast cereals marketed under the same name in 2013 and 2020 (*n* = 134), stratified by year of data collection.

Nutrient Criteria	2013 (Median (IQR))	2020 (Median (IQR))	2013 (Mean ± SD)	2020 (Mean ± SD)	*p*-Value
Energy (kJ)	1590 (1530–1630)	1600 (1540–1640)	1589.8 ± 131.9	1597.6 ± 114.1	0.331
Protein (g)	9.6 (7.8–12.1)	10.1 (8.1–12.4)	10.1 ± 3.2	10.4 ± 3.0	0.036 *
Total Fat (g)	3.4 (1.6–8.4)	4.35 (1.8–8.8)	5.4 ± 5.2	5.9 ± 5.0	0.012 *
Saturated Fat (g)	0.9 (0.4–1.5)	1.0 (0.5–1.6)	1.1 ± 1.0	1.2 ± 1.0	0.205
Carbohydrate (g)	67 (58.1–74.5)	65.55 (57.5–71.8)	67.0 ± 12.1	65.8 ± 11.4	0.000 *
Sugars (g)	17.3 (7.9–24.8)	15 (7.1–22.3)	16.7 ± 11.1	15.1 ± 10.0	0.000 *
Dietary Fibre (g)	9.7 (7.3–11.6)	9.2 (6.9–11.4)	9.9 ± 5.2	9.6 ± 5.3	0.216
Sodium (mg)	167.5 (23–355)	169.5 (16–300)	202.7 ± 186.6	171.4 ± 153.3	0.000 *

* Statistically significant *p* < 0.05.

**Table 5:** Frequency of breakfast cereals displaying nutrition content and health claims in 2013, 2015, 2018, and 2020.

	2013 *n* (%)	2015 *n* (%)	2018 *n* (%)	2020 *n* (%)	Change 2013–2020
**Nutrition Content Claim**					
Plant-based	17 (6)	59 (14)	102 (23)	177 (33)	941%
Protein	27 (10)	77 (18)	81 (18)	161 (30)	496%
Sugar	22 (8)	46 (11)	73 (16)	128 (24)	482%
Gluten Free	27 (10)	44 (10)	69 (15)	92 (17)	241%
Whole Grain	132 (47)	249 (58)	235 (52)	283 (52)	114%
Dietary Fibre	168 (59)	292 (68)	292 (65)	353 (65)	110%
Vitamin/mineral	76 (27)	70 (16)	68 (15)	86 (16)	13%
Sodium	51 (18)	109 (25)	46 (10)	48 (9)	−6%
Fat	66 (23)	70 (16)	44 (10)	60 (11)	−9%
Glycemic index	26 (9)	33 (8)	22 (5)	21 (4)	−19%
**General Level Health Claim**					
Dietary Fibre	7 (2)	3 (1)	36 (80	56 (10)	700%
Iron	1 (0)	1 (0)	13 (3)	15 (3)	1400%
Protein	0	0	11 (2)	10 (2)	NA
**High Level Health Claim**					
B-Glucan and cholesterol	18 (6)	12 (3)	23 (5)	32 (6)	78%

**Table 6:** Comparison of nutrients in whole grain and non-whole grain breakfast cereals (per 100 g) (median (IQR)) in 2020.

Nutrient Criteria	Whole Grain * (*n* = 364)	Non-Whole Grain (*n* = 179)	*p*-Value
Energy (kJ)	1640 (1590–1760)	1650 (1587.5–1885)	0.149
Protein (g)	10.9 (9.3–12.5)	7.8 (6.9–12.3)	0.000 ^α^
Total Fat (g)	10 (6.3–14.5)	4 (1.5–16.9)	0.006 ^α^
Saturated Fat (g)	1.7 (1.2–2.4)	1.0 (0.5–3.1)	0.021 ^α^
Carbohydrate (g)	58.7 (54.9–66.2)	66.4 (48.7–83.2)	0.013 ^α^
Sugars (g)	12.8 (5.8–18)	14.9 (9.5–25.3)	0.000 ^α^
Dietary Fibre (g)	9.2 (7.4–11.3)	7.9 (2.7–12.4)	0.009 ^α^
Sodium (mg)	27 (8–152)	204 (49–340)	0.000 ^α^

* Based on eligibility for registration with Grains & Legumes Nutrition Council (GLNC)’s Code of Practice for Whole Grain Ingredient Content Claims (≥8 g per manufacturer serve). Nut and/or seed-based cereals excluded (*n* = 30). ^α^ Statistically significant *p* < 0.05.

## Data Availability

All data for this study is contained within the article.
